# Medical Notes and Comments

**Published:** 1878-04

**Authors:** Charles A. Wall


### Medical Notes and Comments.

By Charles A. Wall, B. S.

The January number of the Virginia Medical Monthly comes to us enlarged and enriched by the transactions of the Medical Society of Virginia for 1877. This is a mode of publication that is to be commended and other medical bodies would do well to copy it, since in this way a larger number of readers can be reached and influenced than when they are put forth in pamphlet form. The transactions comprise 192 pages which are valuable from begining to end. We have not the space to name the various papers presented, all of which are worth careful reading, but will only call attention to the *Report on advances in Hygiene and Public Health*, by L. S. Joynes, M. D., as an able résumé of the present knowledge upon this interesting and important subject. It shows signs of close research and many of its conclusions, especially in reference to the experimental introductions of the Eucalyptus into the Southern States, deserve to be followed. This number of the Monthly also contains an accurate engraving of Dr. Lewis A. Sayre, with a brief biography of this well known surgeon.

A small pamphlet of some eighty pages, containing the proceedings of the fourth annual meeting of the Medical Society of the State of Oregon has been received. It consists of the Minutes of the meeting, the address of the president, two papers and the constitution of the society.

The transactions of the Medical Society of the County of Erie, for 1877, contains nothing of permanent interest except the action taken upon the death of Drs. Winne, Hadley and Ayer, abstracts of which have appeared in this Journal.

Studies in Pathological Anatomy, by Francis Delafield, M. D. No. I. February, 1878. New York: Wm. Wood & Co. This is the title of a new work to be published monthly, each paper consisting of two or more plates with accompanying text. The first twelve will include the pathological anatomy of pleurisy, of peritonitis and of meningitis. The first number has three full paged plates drawn by the author and represent: plate 1. flat connective tissue cells, plates 2 and 3, branching connective tissue cells, with three pages of descriptive text. Dr. Delafield has already done noble work in this field and we tender him our very best wishes for the success of his new venture.

Dr. Wm. Goodell presented as his address on retiring from the presidency of the Philadelphia County Medical Society, a sketch of the life and writings of Louyse Bourgeois, which will be found readable and of more than passing value. He gives us a slight glance of the state of the profession in the sixteenth century, and brings out in strong relief, its relations to the royal household of France.

Prof. L. P. Yandell, Jr., M. D., in an able paper upon Malaria and Stuma read before the American Dermatological Association and printed in the *American Practitioner*, Jan., 1878, claims for Malaria and Stuma a place, as cause of skin diseases, that is radical and extreme. He says: "I hold that in Malaria we find the chief source of acute skin diseases, and that to scrofula most of the chronic skin diseases may be traced; and that the more inveterate examples of either class are commonly due to a coêxistence of these two causes. And, furthermore, that the favorable or unfavorable course and termination even of the exanthemata are largely influenced by the presence or absence of scrofula and malaria in the patients." He first endeavors to prove that malaria is everywhere present; then gives pathological and therapeutical evidences of the malarial origin of acute skin diseases; and lastly, the evidences of the influence of stuma upon chronic cases, are discussed. The paper brings forward some new ideas that are of value and should be acted upon in many instances. That the conclusion is established, that malaria and stuma are the chief causes of skin diseases, is much to be doubted and time, as it brings clinical facts to bear, will, alone, be able to decide the truth of the statement.

Dr. J. V. Shoemaker calls attention to the uses of the bath in the treatment of skin diseases. In the valedictory address to the class on diseases of the skin at the Philadelphia School of Anatomy and Surgery, he says: "The bath not only cleanses the body, but preserves the health, prevents and assists in the cure of disease by draining from the skin the impurities that have been cast out by the system." He shows the importance of the function of the skin and the manner in which extra labor is thrown upon the lungs and other viscera when the skin is unhealthy. All varieties of the bath are described and their use as indicated, and he ends with the wish that public baths might be established in every city.

On the Recognition and management of the Gouty state in Diseases of the Skin, is the title of a paper published in the *American Practitioner*, Nov., 1877, by L. Duncan Bulkley, A. M., M. D., in which he brings forward many points to show that the effect of gout upon skin diseases, has not been as carefully studied as it deserves. The following by the same author present many new facts. On the so-called Eczema Marginatum of Hebra; read at the first annual meeting of the American Dermatological Association; reprint from the *Chicago Medical Journal and Examiner*, Nov. 1877. Are Eczema and Psoriasis local diseases of the skin or are they manifestations of constitutional disorders? Read before the International Medical Congress, 1876.

On certain points relating to the nature and treatment of Lupus, by Henry G. Piffard, A. M., M., D. Read before the Medical Society of the State of New York, 1877, is a paper that will interest many.

We are in receipt of the reprint from the *British Medical Journal*, Dec. 1877, of a paper on Battey's Operation, by J. Marion Sims, A. M., M. D. Dr. Battey and his method of removing the ovaries in cases imperatively demanding this radical procedure, are well known to American readers, and we congratulate him upon having so talented an advocate as Dr. Sims to present his case before the profession in England. The operation has been performed twenty-eight times since it was first proposed by Dr. Battey in 1872; twelve being done by the originator, while Dr. Sims stands next with seven cases. This operation is now justified by its success and perhaps it is well that it should take the name, as Dr. Sims suggests, of its originator, and as we have Syme's, Peragoff's and Graeffe's operations so let this be known as Battey's operation.

Dr. W. H. Wathen has reported in the *Louisville Medical Record* six interesting cases which he has reprinted under the title of *Clinical Gynæcology*, the most important being case first, on Utero-Tubal pregnancy, with dropsy of the villi of the chorion. We do not remember to have seen the record of a similar case.

Dr. Easley reprints from the Feb. number of *Richmond and Louisville Medical Journal* a case of Intra-Uterine Submucous Fibroid. The conclusions arrived at in this article are sound and show that the author has carefully considered the subject in all its bearings.

Professor Lewis A. Sayre, M. D., gives a succinct history of the plan of treatment of Pott's Disease by suspension and the use of plaster of Paris bandage, in which he clears himself from the charge of plagiarism of which he was so unjustly accused. Dr. Sayre has given to others full credit in all of his publications, so far as we could judge, even before we received this latest proof, and we hope that every one will be satisfied.

Circular No. 10. Approved Plans and Specifications for Post Hospitals, Surgeon General's Office, Washington, Oct. 20, 1877. This work consists of ten plates with descriptions and specifications to go with them. The plans are for regulation Post Hospitals of from six to twenty-four beds, and are designed for various localities in accordance with the surrounding circumstances. They are four in number the first two plans consisting of a central administration building and wards, as wings, one story in height. Plan 3 is for malarial regions and the wards are therefore placed on the second story. Plan 4 is for a provisional hospital of 6 or 8 beds to be built of logs or other material at hand. These plans are modern in every particular.

We have not the space to notice many pamphlets which lie before us, and would refer the reader to the proper head in this number where the titles are given in acknowledging their receipt.

'E. A. Carpenter in *New Remedies*,' "a friend of mine, an English chemist, laughs at the idea of bicarbonate of sodium being a new treatment for burns. He says, that in England, twelve years ago, it was known to all working chemists." Many cases have been reported lately of the use of this remedy, and if it does half that is claimed, it is a grand step forward in the treatment of these painful accidents. We hope that all who may have experience will inform the profession.

The *Philadelphia Druggist and Chemist*, is the name of a new monthly, edited by C. C. Vanderbeck, M. D., Ph. D., devoted to Materia Medica, Chemistry, Therapeutics and the Collateral Sciences. Each number consists of 35 closely printed pages containing many things of equal importance to the Physician, Druggist and Chemist. The price is only $1.50 per year, to be forwarded to the Editor at No. 1011 Walnut St., Philadelphia.

The *Western Lancet* has changed hands, George Hewston, A. M., M. D., and James Simpson, M. D., assuming editorial control. The March number, owing to delay in issuing the Journal, constitutes No. 1 of Vol. VII, and in consequence, the volume will hereafter commence with March instead of January. We bespeak for the new Editors a cordial reception.

The January number of the *St. Louis Medical and Surgical Journal*, has taken on a new cover with a change of Editors, Drs. J. F. Rumbold, and H. Christopher succeed Drs. Edgar and Dean.

Dr. Priest has withdrawn from the *Toledo Medical and Surgical Journal.* Drs. Chapman and Waddle remain editors as formerly and have associated with them Drs. Collamore and Currey.

Drs. Miles and Bramble have closed their connection with the *Cincinnati Medical News*, Drs. Thacker and Reed being the sole proprietors and editors; the former will act as manager of all business.

Macmillan & Co., London and Cambridge, and 22 Bond Street, New York, have sent forth a prospectus of a new Journal, soon to be started. The title page reads as follows: The Journal of Physiology, edited with the co-operation in England of Prof. A. Gamgee, F. R. S., of Manchester; Prof. W. Rutherford, F. R. S., of Edinburg; Prof. J. B. Sanderson, F. R. S., of London; and in America of Prof. H. P. Bowdich of Boston; Prof. H. N. Martin of Baltimore; by Dr. MICHAEL FOSTER, F. R. S., of Trinity College, Cambridge. It will be published in parts not at rigidly fixed times, but according to the supply of material. Four or six parts will form a volume of about 500 pages, at £1, 1s or $5 25 (gold.) The first part will soon appear.

Dr. W. L. NICHOL has retired from the editorship of the *Nashville Journal of Medicine and Surgery*, Dr. C. S. Briggs continues as sole editor.

IT may be of interest, to many, to know that a house for those having acquired the opium habit, has lately been started. It is located at Brooklyn, N. Y., is called Parrish Hall, and as will be seen by its advertisement, is exclusively devoted to the welfare of those unfortunates who may place themselves within its influence.

———:o:———

WE take pleasure in calling the attention of the profession to that important remedial agent, Lactopepsin, so highly recommended by many leading physicians. Composed as it is of sugar of milk, pepsin, pancreatine and other digestive fluids, we see no reason why it should not be extremely useful in all cases where indicated.

ERRATA. March number, page 296, second line, instead of Boston read Barton in Dr Mursick's correspondence; and in Dr. Hamilton's article on page 310, line 13 read "pouring oil into each other's, etc.," instead of "pouring into each other's, etc."

